# Interleukin-12 Plasmid DNA Delivery by *N*-[(2-Hydroxy-3-trimethylammonium)propyl]chitosan-Based Nanoparticles

**DOI:** 10.3390/polym14112176

**Published:** 2022-05-27

**Authors:** Ali Dehshahri, Bahman Khalvati, Zahra Taheri, Farshad Safari, Reza Mohammadinejad, Abolfazl Heydari

**Affiliations:** 1 Pharmaceutical Sciences Research Center, School of Pharmacy, Shiraz University of Medical Sciences, Shiraz P.O. Box 7146864685, Iran; dehshahria@sums.ac.ir (A.D.); zahra.taheri436shz@yahoo.com (Z.T.); 2 Medicinal Plants Research Center, Yasuj University of Medical Sciences, Yasuj P.O. Box 7591994779, Iran; bahman.khalvati@gmail.com (B.K.); safarii.farshad@gmail.com (F.S.); 3 Student Research Committee, Shiraz University of Medical Sciences, Shiraz P.O. Box 7146864685, Iran; 4 Research Center of Tropical and Infectious Diseases, Kerman University of Medical Sciences, Kerman P.O. Box 76187-47653, Iran; 5 Polymer Institute of the Slovak Academy of Sciences, Dúbravská cesta 9, 845 41 Bratislava, Slovakia

**Keywords:** quaternized chitosan, polyplex, nanoparticles, interleukin-12, gene delivery

## Abstract

Cationic polysaccharides are capable of forming polyplexes with nucleic acids and are considered promising polymeric gene carriers. The objective of this study was to evaluate the transfection efficiency and cytotoxicity of *N*-[(2-hydroxy-3-trimethylammonium)propyl] chitosan salt (HTCS), a quaternary ammonium derivative of chitosan (CS), which benefits from non-ionizable positive charges. In this work, HTCS with a full quaternization of amino groups and a molar mass of 130,000 g·mol^−1^ was synthesized to use for delivery of a plasmid encoding the interleukin-12 (IL-12) gene. Thus, a polyplex based on HTCS and the IL-12 plasmid was prepared and then was characterized in terms of particle size, zeta potential, plasmid condensation ability, and protection of the plasmid against enzymatic degradation. We showed that HTCS was able to condense the IL-12 plasmid by the formation of polyplexes in the range of 74.5 ± 0.75 nm. The level of hIL-12 production following the transfection of the cells with HTCS polyplexes at a C/P ratio of 8:1 was around 4.8- and 2.2-fold higher than with CS and polyethylenimine polyplexes, respectively. These findings highlight the role of HTCS in the formation of polyplexes for the efficient delivery of plasmid DNA.

## 1. Introduction

Gene therapy has attracted great attention during the last decades for the treatment or prevention of various diseases [1,2]. Cytokine-mediated immunotherapy has provided a great opportunity for the treatment of various diseases, including cancer. Interleukin-12 (IL-12) is one of the most investigated cytokines for cancer therapy [3]. This pro-inflammatory heterodimer cytokine is mainly secreted by antigen-presenting cells and is composed of two subunits linked via disulfide bonds to form a biologically active heterodimer. The antitumor effect of IL-12 is mainly mediated by CD8^+^ T cells and natural killer (NK) cells [4]. IL-12 has shown antitumor effects in several in vitro and in vivo investigations [3]. The potent antitumor properties of IL-12 in animal models and its robust immune stimulatory effects in human clinical trials encouraged various researchers to focus on the development of this cytokine as a potential cancer therapeutic agent [5]. However, great disappointment occurred following the systemic administration of recombinant human IL-12 proteins in human clinical trials. Severe systemic toxicity following the injection of IL-12 proteins into patients led to on-study deaths and reduced enthusiasm for the future human application of this cytokine as a drug candidate [3]. The main reasons for failed efforts to use IL-12 as a systemic therapeutic agent in cancer therapy are associated with the systemic overproduction of IFN-γ and insufficient delivery of the cytokine to the tumor microenvironment as its precise site of action. Therefore, the local delivery of a plasmid encoding the IL-12 gene to tumor sites has been considered as an alternative strategy [6]. This new strategy can augment the local antitumor effects of IL-12 while reducing its systemic toxic properties [7,8]. One of the major breakthroughs in the local administration of the IL-12 gene was observed following the intratumoral injection of IL-12 plasmid formulated with nanoparticles [9]. The intratumoral injection of the plasmid resulted in robust antitumor immunity. In this regard, various delivery systems have been designed to transfer this plasmid to the precise site of action [10].

The approval of novel therapeutic modalities for gene therapy has led researchers to seek efficient and safe delivery systems enabling the transfer of genetic materials to the site of action. The main approach for gene delivery is the application of viral-based delivery systems. Despite their high efficiency for gene delivery, major concerns have limited their wide clinical application [11]. These concerns include probable immunogenicity, oncogenicity, and limited capacity for gene delivery, as well as expensive and laborious scale-up procedures. Hence, there is growing attention to non-viral gene delivery methods, including polymer-based gene carriers involving polyplex formation [12]. These complexes may contain plasmid DNA, siRNA, or mRNA. Polycationic compounds can be considered as highly tailored gene delivery platforms, encouraging researchers to produce a new library of polycationic compounds for potential application in human trials. Several polycationic polymers have been investigated for non-viral gene delivery, including polyethylenimine (PEI) [13,14], polypropylenimine (PPI) [15], polyamidoamide (PAMAM) [16], and chitosan (CS) [17,18,19,20]. In recent decades, considering the safety, cytocompatibility, and gene transfer efficiency, CS has been considered one of the most investigated non-viral polycationic compounds for gene delivery [17,18,19,21]. Despite the potential for gene transfer purposes, CS has not made great achievements in human clinical trials, which might be due to its insolubility and the low stability of CS-based particles at physiological pH. Note that the amino groups of CS are deprotonated at pH higher than 6.4, which is above its pKa value, and consequently, particles based on CS can dissociate into separate components at physiological pH 7.4 [22]. To address this challenge, various strategies have been employed to improve the solubility of CS at physiological pH, which could result in the higher stability of complexes, improved transfection efficiency, and targetability [23,24,25,26,27]. The selection of suitable CS derivatives for gene delivery depends on several factors, including molecular mass, the degree of deacetylation, the modification degree of CS derivatives [28], and also the pH independence of the prepared complex. Among the CS derivatives, converting the ionizable CS to non-ionizable CS derivatives by introducing quaternary ammonium groups to the CS backbone is the most investigated strategy tested for gene and drug delivery. In this regard, N,N,N-trimethyl-CS, N,N,N-triethyl-CS (TEC), N,N,N-dimethylethyl-CS (DMEC), and *N*-[(2-hydroxy-3-trimethylammonium) propyl] CS salt (HTCS) have been synthesized and used for gene delivery [29,30,31].

In this work, we opted to prepare quaternized CS, i.e., HTCS, with as high as possible degree of quaternization and a molar mass of 130,000 g·mol^−1^. This CS derivative contains a non-ionizable quaternary ammonium group and is soluble at physiological pH. Then, the ability of HTCS to deliver a plasmid encoding the IL-12 gene with the formation of polyplexes was investigated. The polyplexes were characterized in terms of particle size, zeta potential, complexation ability, and the capability to protect plasmids against enzymatic degradation. Finally, we evaluated the transfection efficiency and cytotoxicity of polyplexes prepared from the interaction between HTCS and the IL-12 plasmid.

## 2. Materials and Methods

### 2.1. Materials

Commercial chitosan (CS), 95/20, with a degree of acetylation (DA) of 2.5% (by elemental analysis (EA 1108 CHNO analyzer, Fisons Instruments, Ipswich, UK)) and a molar mass of 40,000–150,000 g mol^−1^ (indicated by the manufacturer) was purchased from Heppe Medical Chitosan (Halle, Germany). Branched polyethylenimine (PEI, average molar mass of g·mol^−1^), *N*-[2-hydroxythyl] piperazine-N0-[2-ethanesulfonicacid] (HEPES), and 3-(4,5-dimethylthiazol-2-yl)-2,5-diphenyltetrazolium bromide (MTT) were purchased from Sigma-Aldrich (Saint Louis, MO, USA). Human IL-12 plasmid (pUMVC3-hIL12) was obtained from Aldevron (Madison, WI, USA). Human IL-12 (p70) ELISA Kit was purchased from BD Bioscience (Heidelberg, Germany). EndoFree Plasmid Mega Kit was obtained from Qiagen (Hilden, Germany). DNase I and DNA ladder 1 kb were purchased from Cinnagen (Tehran, Iran). Fetal bovine serum (FBS) and Dulbecco’s Modified Eagle’s Medium (DMEM) were purchased from Gibco (Gaithersburg, MD, USA). All solvents and chemicals were obtained from Sigma-Aldrich.

### 2.2. Quaternized Chitosan Preparation

Quaternized CS, i.e., *N*-[(2-hydroxy-3-trimethylammonium) propyl]chitosan salt (HTCS), was synthesized according to the procedure described by Heydari et al. [32]. In short, 5.0 g of CS (30.3 mmol of glucosamine) was dispersed in 100 mL of water and stirred for 30 min at 80 °C. Then, 32.5 mL (242.4 mmol) of glycidyltrimethyl ammonium chloride (GTMAC, ≥90%, Sigma-Aldrich, Saint Louis, MO, USA) was added in six equal volumes in 1 h intervals, and the reaction proceeded at 80 °C for 16 h. The resulting solution was diluted with water and precipitated in an excess of cold acetone. The polymer was extracted using a Soxhlet extractor in ethanol and acetone and then dried in the oven at 50 °C for 24 h. The degree of quaternization (DQ) was 116 %, determined by ^1^H NMR, and the weight-average molar mass was 130,000 g·mol^−1^, measured following the protocols recommended by us previously [32].

#### 2.2.1. Determination of the Buffering Capacity 

The polymers were dissolved in 10 mL of 150 mmol·L^−1^ NaCl with a pH of 2.0 (adjusted with 0.1 mol·L^−1^ HCl) to obtain a final concentration of 3 mmol·L^−1^; then, the pH of the solution was brought to 3.0 with 0.1 mol·L^−1^ NaOH. Each solution was titrated at 25 °C by incrementally adding 0.1 mol·L^−1^ NaOH using a Hanna automatic titrator (Model HI901C; Hanna Instruments, Woonsocket, RI, USA). The pH values of all solutions were measured by using a HI 1131B electrode (Hanna Instruments, Woonsocket, RI, USA) after each addition. The following protocol was applied for titration: dosing type: linear, 0.1 mL; endpoint mode: fixed, pH 11.50; pre-titration volume: 0 mL; pre-titration time: 0 s; measurement mode: time increment, 30 s; and volume flow rate: 25 mL·min^−1^. NaCl at a concentration of 150 mmol·L^−1^ was used as a control. Buffering capacity of samples was calculated using Equation (1) as described previously by others [33,34].

(1) $$\mathrm{Buffering}\;\mathrm{capacity}=\frac{dn_{\mathrm{NaOH}}}{d\mathrm{pH}}$$

where *n*_NaOH_ is the number of moles of added base. To ensure repeatability, each test was repeated three times.

#### 2.2.2. Water Solubility

The solubility of CS and HTCS in an aqueous medium at pH from 3 to 12 was determined according to the published procedure [35,36]. Briefly, 30 mg of CS or HTCS was added to 3 mL of water, and the pH of the solution was adjusted to 3.0 with 0.1 mol·L^−1^ HCl. Then, pH was increased to 13 using 1 mol·L^−1^ NaOH. The transmittance of the polymer solutions was recorded on a UV–visible spectrophotometer (at 600 nm, UV-16 50PC, Shimadzu, Kyoto, Japan).

### 2.3. Propagation and Purification of Plasmids

The plasmid (pUMVC3-hIL12) was propagated in selective Luria–Bertani (LB) medium containing a selective antibiotic (kanamycin) following the transformation into *Escherichia coli* bacterial strain DH5α. The purification of the plasmid was carried out using Qiagen EndoFree Mega Plasmid Kit (Qiagen GmbH, Hilden, Germany) according to the manufacturer’s instructions. The concentration and purity of the plasmids were assessed by ultraviolet (UV) spectrophotometer. In addition, the size and integrity of the plasmids were confirmed using agarose gel electrophoresis.

### 2.4. Polyplex Preparation

Polyplexes were prepared by adding 50 μL of a solution containing various concentrations of polymers (CS, HTCS, and PEI) to the same volume of plasmid DNA (40 μg·mL^−1^) in HEPES-buffered glucose solution (HBG buffer, 20 mmol·L^−1^ HEPES and 5% glucose) at pH 7.2. Then, the prepared formulations were incubated at ambient temperature for 5–10 min in order to form stable polyplexes. A carrier/plasmid (C/P) ratio (*w*:*w*) was used to define the composition of polyplexes, where C is the weight of polymers and P represents the weight of plasmid DNA in the final formulations.

### 2.5. Biophysical Characterization of Polyplexes

#### 2.5.1. Determination of Plasmid DNA Condensation by Gel Retardation Assay

To assess the plasmid DNA condensation by PEI (as control), CS, or HTCS, a gel retardation assay was performed. Polyplexes were prepared as described earlier (Section 2.4) at different C/P ratios ranging from 0.5:1 to 8:1. Then, 10 μL of each polyplex formulation was loaded onto 1% agarose gel. A UV transilluminator (Perkin-Elmer, Foster City, CA, USA) was employed to visualize plasmid DNA bands [37].

#### 2.5.2. Plasmid Protection by DNase I Protection Assay

To assess the capability of PEI (as control), CS, or HTCS to protect plasmid DNA against enzymatic degradation, a DNase I protection assay was carried out as described in the literature [38]. The polyplex formulations were prepared as described in Section 2.4 and mixed with 1 mL of DNase I or PBS in DNase/Mg^2+^ digestion buffer (50 mM Tris–Cl, pH 7.6 and 10 mmol·L^−1^ MgCl_2_). The plasmid bands were analyzed by agarose gel electrophoresis at 70 V for 1 h.

#### 2.5.3. Measurements of Particle Size and Zeta Potential 

To measure the sizes and zeta potentials of the polyplexes, the desired amounts of PEI, CS, or HTCS dissolved in HBG buffer (pH 7.4) were added to the plasmid DNA solution prepared in the same buffer. The sizes and zeta potentials of the polyplexes were measured by dynamic light scattering (DLS) and laser Doppler velocimetry (LDV), respectively, using a Horiba Sz-100 nanoparticle size analyzer (Kyoto, Japan). The measurements were carried out using automatic mode, and the results are presented as mean ± SD, n = 3. Each mean represents the average value of 30 measurements.

### 2.6. Biological Studies

#### 2.6.1. Cell Culture and Cell Viability Assay

Human HepG2 hepatocellular carcinoma cells (NCBI C158, Tehran, Iran) and breast MCF-7 adenocarcinoma (NCBI C135, Tehran, Iran) cells were obtained from National Cell Bank of Iran (Pasteur Institute, Tehran, Iran) and maintained at 37 °C and 5% CO_2_ in DMEM supplemented with 10% FBS, streptomycin at 100 μg·mL^−1^, and penicillin at 100 U/mL. One day before starting toxicity studies, cells were seeded at a density of 1 × 10^4^ cells/well in 96-well trays for 24 h. The cytotoxicity of serial dilutions of PEI, CS, and HTCS at concentrations ranging from 2 to 100 μg·mL^−1^ was measured using the MTT assay. Ten microliters of each concentration was added to the wells and incubated at 37 °C for 4 h. Then, the medium was replaced with 100 μL of fresh medium. Forty-eight hours later, the medium was aspirated, and the MTT solution (5 μg·mL^−1^) was added to each well and incubated for 2 h. The formazan crystals were dissolved in 100 μL/well dimethyl sulfoxide (DMSO); the absorbance was measured by an ELISA reader at 590 nm, and background was corrected at 630 nm according to our reported procedure [39]. Data are presented as mean ± SD; *n* = 3.

#### 2.6.2. In Vitro Plasmid Delivery and Evaluation of IL-12 Expression

To evaluate the ability of polymers to transfer the plasmid encoding the human IL-12 gene, polyplex formulations were prepared at C/P ratios of 0.5:1, 4:1, and 8:1. Then, 10 μL of each polyplex formulation (equivalent to 200 ng plasmid DNA) was added to the wells and incubated under the same conditions as described for the cytotoxicity assay. After 48 h, hIL-12 quantification was carried out by collecting the cell supernatants. The level of hIL-12 in the cell supernatants was measured by human IL-12 (p70) ELISA kit (BD Bioscience, Heidelberg, Germany) according to the manufacturer’s protocol. The results of hIL-12 expression were normalized and are presented as pg/mL/seeded cells, as described by Lotfipour et al. [40]. Cells treated with no plasmid or polyplex were considered controls.

### 2.7. Statistics

Data are presented as the mean ± SD. The statistical significance was determined using Student’s *t*-test, and *p* values of ≤0.05 were considered significant.

## 3. Results and Discussion

The principal goal of this study was to design a polyplex delivery system based on non-ionizable polycationic polysaccharides for nucleic acid delivery. We modified all free amino groups of CS by attaching quaternary ammonium groups to the polymer backbone through a relatively simple chemical reaction for HTCS preparation [32]. Then, the cationic CS derivative was used to prepare a polyplex through the formation of a complex between the positively charged CS and the negatively charged nucleic acid. This type of interaction could lead to the formation of polyplexes with a desirable performance under physiological conditions and a favorable size protecting plasmid DNA from enzymatic degradation in extra- and intracellular environments.

### 3.1. Quaternized Chitosan Properties

**Figure 1:**
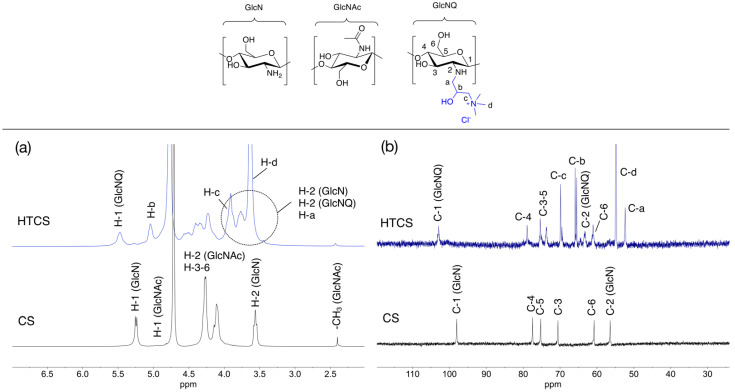
(**a**) ^1^H NMR (400 MHz) and (**b**) ^13^C NMR (100 MHz) spectra of CS (10 mg·mL^−1^) and HTCS (20 mg·mL^−1^) in D_2_O/DCl at 65 °C. The chemical structures of sugar units are inserted.

The synthesized HTCS was characterized by NMR analysis (Figure 1) as described in detail by us previously [32]. The ^1^H NMR and ^13^C NMR spectra of CS and HTCS are shown in Figure 1a,b with highlighted characteristic proton and carbon signals. These spectra confirm the presence of the pendant quaternary ammonium moieties in HTCS.

#### 3.1.1. Measurement of Buffering Capacity

**Figure 2:**
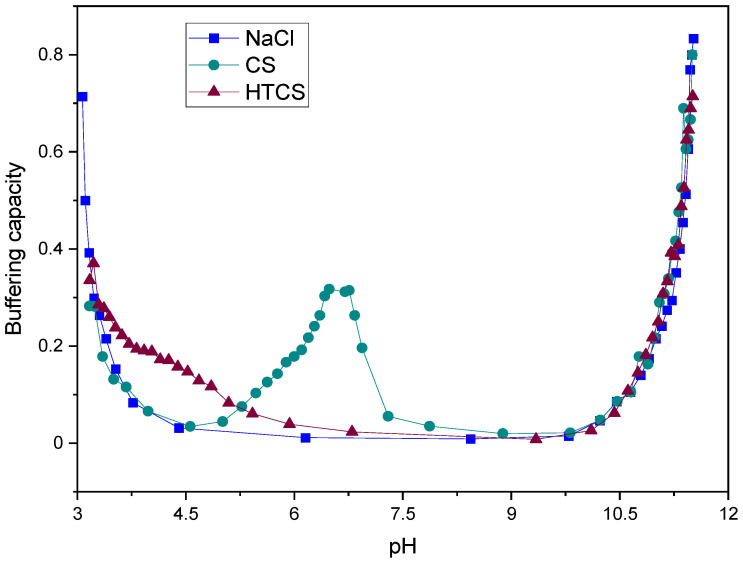
Buffering capacity curves of NaCl, CS, and HTCS in pH ranging from 3.0 to 11.0.

The buffering capacity is expressed as the change in pH upon the addition of a certain amount of base. The buffering capacity of CS and HTCS was plotted graphically against pH in a defined range, which was obtained by the acid–base titration method (Figure 2). In order to fully protonate and deprotonate pH-sensitive charges on these polymers, the experiment was carried out at a pH ranging from 3 to 11, which are below and above the pKa values of non-modified (pKa 6.1) and modified (pKa 4.1) sugar units [41]. In principle, CS has one titratable proton, which is the primary amine on the sugar unit [42,43]. Figure 2 shows the charge neutralization of the CS solution, which gives only one maximum peak in the buffering capacity curve located at pH 6.4, related to equivalence points in the acid–base titration. On the other hand, the attachment of quaternary ammonium groups to the CS backbone shifted the buffering capacity of the polymer from pH 6.4 to pH 4.1. This is due to the conversion of all of the free amino groups (primary amines) on the CS structure to secondary amines during the HTCS synthesis.

The proton sponge effect is the main mechanism by which polycationic polymers induce the early escape of the polyplexes from endosomes [44]. The induction of early escape from endo/lysosomal vesicles is associated with the presence of various amine groups in the structure of polycations. These amines act as a proton sponge and prevent changes in the endosomal pH following the influx of protons into the compartments. In other words, the presence of amines in polycations results in the capture of protons in endosomal vesicles. Following the influx of protons and chloride ions, the influx of water results in endosomal swelling and rupture. This early escape prevents the degradation of nucleic acid materials by various degrading enzymes, which act in the acidic environment of lysosomes. It has been shown that the buffering capacity under acidic conditions acts as a driving force for an osmotic burst of endosomes, resulting in the early escape of polyplexes to the cytosol. Hence, the quaternization of CS resulted in a higher buffering capacity at lower pH by shifting the corresponding values from pH 6.4 to pH 4.1. This is essential to induce early escape from endo/lysosomal compartments and consequently may be associated with higher transfection efficiencies.

#### 3.1.2. Water Solubility

**Figure 3:**
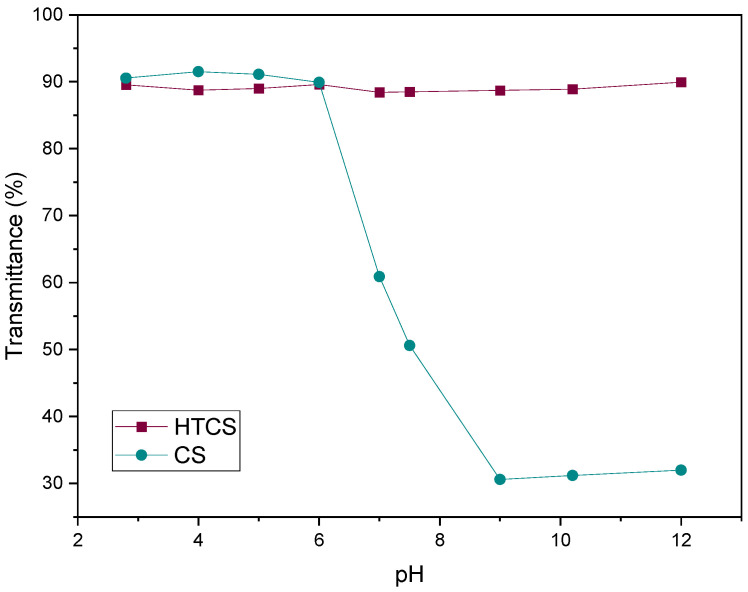
The water solubility of CS and HTCS at pH ranging from 3 to 12. The transmittance of the polymeric solution was measured with UV-vis.

Figure 3 shows the transmittance of CS and HTCS in an aqueous medium at pH ranging from 3 to 12. The polymers were considered insoluble when the transmittance of the solution was less than 90%. CS is insoluble at pH below around 7.4, i.e., physiological pH, which is due to the deprotonation of the positively charged nitrogen groups. In contrast, the introduction of the quaternary ammonium group to the CS backbone makes the solubility of HTCS independent of pH. The reason for this is that the degree of dissociation of the ionic groups on the quaternary ammonium group is pH-independent over a wide pH range, highlighting its strong polycationic character.

### 3.2. Binding Affinity of CS and Quaternized CS to Plasmid DNA

**Figure 4:**
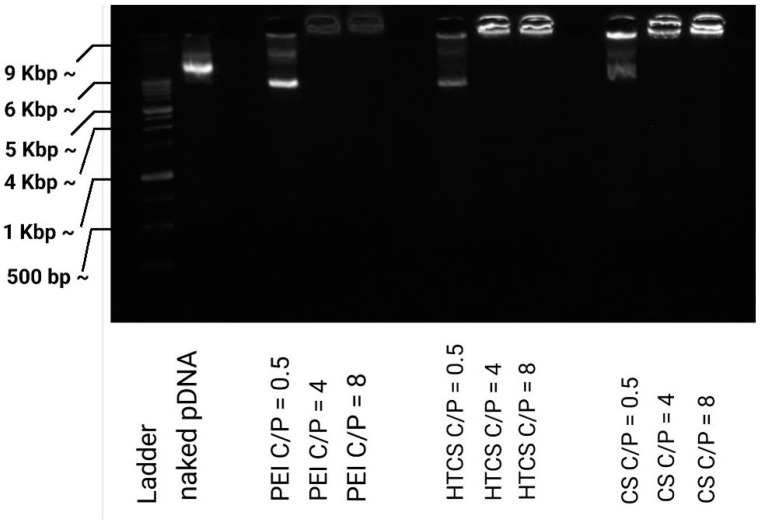
Plasmid DNA binding affinity of PEI, CS, and HTCS determined by gel retardation assay at C/P ratios of 0.5:1, 4:1, and 8:1.

The strength of the interaction between polycationic material and plasmid DNA can be determined by a gel retardation assay, in which the migration of the plasmid in the gel can be retarded due to its association with the polycationic surfaces of the polymers. In this work, the ability of CS, HTCS, and PEI was studied using a gel retardation assay. Figure 4 shows that CS, HTCS, and PEI were able to completely condense the plasmid DNA at C/P ratios of 4 and 8, while the condensation ability was not complete at the lowest C/P ratio of 0.5. On the other hand, the patterns of the condensation ability of CS, HTCS, and PEI were similar (Figure 4). The ladder and naked DNA lanes confirmed the size and integrity of the plasmid, respectively. 

It should be noted that the condensation ability has been shown to have a critical role in transfection efficiency, which has been studied in several investigations [21,45,46,47]. On the one hand, the interaction between the polymer and nucleic acid materials is necessary for polyplex formation, protection against enzymatic digestion, and cell entry via adsorptive endocytosis. On the other hand, nucleic acid materials must be released inside the cell. There are some reports indicating that promoting vector unpackaging may facilitate the accessibility of plasmid DNA to the transcriptional machinery of the cell, leading to higher gene transfer ability [48]. However, there are other controversial observations showing that the formation of tight polyplexes does not necessarily lead to lower transfection efficiency [49]. It seems that the formation of loose polyplexes is not a promising strategy for increasing transfection efficiency since this process is not the rate-limiting step in gene transfer [47]. In our study, the strength of the binding affinity between HTCS and plasmid DNA was similar to that of CS and PEI, considered the gold standard of a polycation-mediated gene delivery system.

### 3.3. Protection of Plasmid DNA against DNase I Digestion

**Figure 5:**
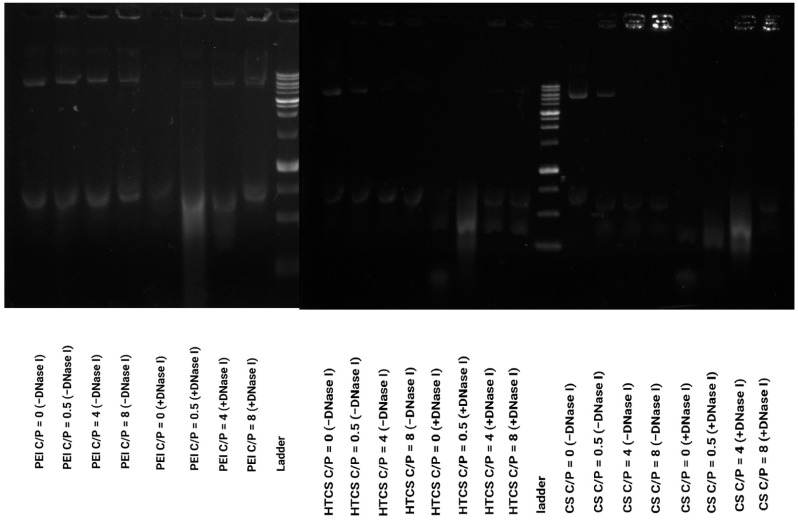
DNase I protection assay for PEI, CS, and HTCS. The polyplexes were treated with DNase I or PBS (negative control).

The electrostatic interaction of polycationic polymers and the plasmid not only leads to the condensation of pDNA and the formation of polyplexes but also protects nucleic acid materials from enzymatic degradation. Naked plasmid DNA is susceptible to enzymatic digestion in the blood circulation and intracellular environment. To measure the protection of plasmid DNA following the formation of the polyplex, a DNaseI digestion assay was carried out. The results of the protection and release assay (Figure 5) demonstrate that the enzymatic treatment of naked pDNA (i.e., without carrier) resulted in the complete degradation of the nucleic acid material. Figure 5 shows that at the lowest C/P ratio, C/P = 0.5:1, the polyplexes formed by CS, PEI, and HTCS could not protect plasmid DNA against enzymatic digestion. At this C/P ratio, the plasmid was completely degraded. On the other hand, partial protection against digestion occurred at C/P ratios of 4:1 and 8:1. These findings reveal that the protection behavior of HTCS is similar to that of CS at all C/P ratios, indicating that the chemical modification of CS did not reduce the protection ability of CS while increasing its binding strength for nucleic acid condensation. Figure 4 shows the complete condensation of plasmid DNA at C/P ratios of 4:1 and 8:1. However, the results of the protection assay show that complete condensation does not lead to complete protection (Figure 5). This might be due to the formation of loose polyplexes in a way that causes some portions of the plasmid to be located on the surface of complexes, making them more accessible to degrading enzymes and consequently increasing enzymatic degradation [50].

### 3.4. Particle Size and Zeta Potential Measurements

The formation of polyplexes with favorable particle size and charge density plays a crucial role in transfection efficiency and cytotoxicity. There are several investigations indicating the impact of particle size on the interaction of polyplexes with biological systems [51,52,53]. It has been shown that clathrin-mediated endocytosis occurs for particles with a size of around 120–150 nm and not higher than 200 nm [54]. Thus, in our study, the polyplex was formed at the highest C/P ratio (C/P = 8:1) and characterized in terms of particle size and zeta potential. Table 1 shows that HTCS formed polyplexes with a size of 74.5 ± 0.75 nm, while the size of CS-based polyplexes was 66.5 ± 0.55 nm with a low polydispersity index (PDI < 0.3). Although the size of HTCS complexes was slightly larger, this difference was not statistically significant (*p* > 0.5). It could be the result of the presence of the pendant quaternary ammonium group of HTCS in comparison with CS. The size for PEI-based polyplexes was 97.5 ± 1 nm under the same conditions, which is in agreement with previous observations [39].

The results of LDV revealed that the zeta potential of HTCS-based polyplexes was higher than that of the polyplexes prepared by CS. This finding highlights that the quaternization of CS during HTCS preparation increased the positive charge density of the corresponding polyplex. Although the formation of polyplexes with higher cationic charge density may result in more toxicity in the cells [55], the zeta potential of HTCS polyplexes was not higher than that of PEI-based complexes, indicating a desirable cationic charge density on the complexes.

### 3.5. Cell Viability Experiments and Gene Transfer Ability

**Figure 6:**
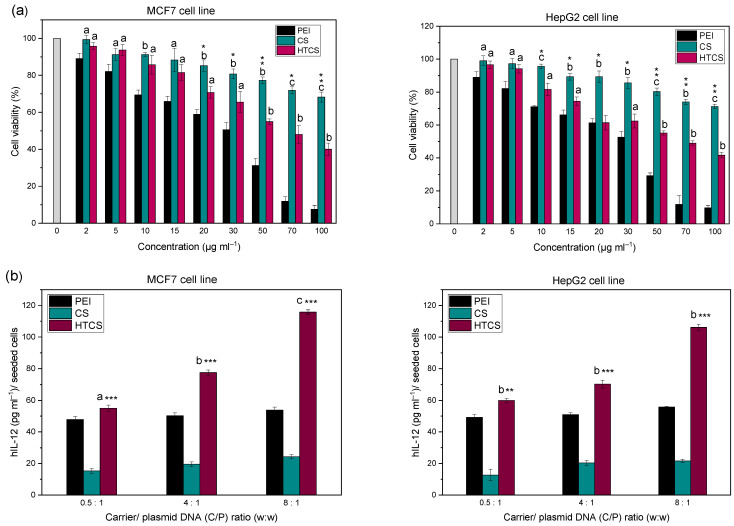
(**a**) Cell viability of MCF-7 and HepG2 cells as a function of concentration of PEI, CS, and HTCS in the medium. ^a^ *p* < 0.05, CS and HTCS compared with PEI at the same concentration; ^b^ *p* < 0.01, CS and HTCS compared with PEI at the same concentration; ^c^ *p* < 0.001, CS and HTCS compared with PEI at the same concentration. * *p* < 0.05, HTCS compared with CS at the same concentration; ** *p* < 0.01, HTCS compared with CS at the same concentration; *** *p* < 0.001, HTCS compared with CS at the same concentration. (**b**) Gene transfer ability of PEI, CS, and HTCS. The levels of hIL-12 in MCF-7 cells and HepG2 cell following treatment with polyplexes at C/P ratios of 0.5, 4, and 8. The level of hIL-12 expression is presented as the concentration of the protein (pg·mL^−1^) per seeded cell. ^a^ *p* < 0.05, CS and HTCS compared with PEI at the same C/P ratio; ^b^ *p* < 0.01, CS and HTCS compared with PEI at the same C/P ratio; ^c^ *p* < 0.001, CS and HTCS compared with PEI at the same C/P ratio. * *p* < 0.05, HTCS compared with CS at the same C/P ratio; ** *p* < 0.01, HTCS compared with CS at the same C/P ratio; *** *p* < 0.001, HTCS compared to CS at the same C/P ratio.

The cytotoxicity of PEI, CS, and HTCS was determined in HepG2 and MCF-7 cells using an MTT colorimetric assay by measuring cell viability values. The cytotoxicity of HTCS was compared to that of PEI and CS at the same concentration (Figure 6a). Figure 6 demonstrates that the viability of both cell lines decreased when increasing the concentration of polycationic compounds. There was concentration-dependent toxicity for all compounds. The toxic effect of PEI at all concentrations was much higher than that of CS and HTCS. At the highest concentration (i.e., 100 μg·mL^−1^), around 70% and 40% viabilities were observed for CS and HTCS, respectively. At the same concentration, the viability of cells treated with PEI was around 10%. Therefore, the cytotoxicity of HTCS was higher than that of CS, while its toxicity was remarkably lower than that of PEI. In the MCF-7 cell line, the IC_50_ of HTCS was 70 μg·mL^−1^, while the IC_50_ of PEI was observed at 30 μg·mL^−1^.

The viability test revealed that the introduction of the quaternary ammonium group to CS increased the toxicity of modified CS in comparison with non-modified CS. However, the toxicity of HTCS was lower than that of PEI. One of the mechanisms of the toxicity of cationic polymers is the high positive charge density on their structure. This charge density leads to the interaction of polycationic compounds with negatively charged components on the cell surface, resulting in the perturbation of the cell membrane [56]. According to the zeta potential measurement, PEI and HTCS polyplexes showed similar levels of charge density. However, they resulted in different levels of cell viability. The results of cell viability revealed that HTCS-induced toxicity was significantly lower than that induced by PEI. This observation highlights that the cationic charge density is not the only factor determining the level of toxicity. Other parameters, such as the molecular weight, shape, and orientation of the polyplexes, may also have an influence on the level of toxic effects of the polyplexes on cells [57,58]. Even at the highest concentration of 100 μg·mL^−1^, HTCS led to a viability of around 40%, whereas the viability of PEI at the same concentration was less than 10%. This may allow researchers to use higher amounts of such polymers for more efficient condensation of nucleic acid materials, while lower toxicity is expected.

In this work, the efficiency of HTCS for IL-12 plasmid delivery was measured in MCF-7 and HepG2 cell lines (Figure 6b). The transfection efficiency with HTCS polyplexes was higher than with CS and PEI at all tested C/P ratios. The highest level of hIL-12 production was achieved by HTCS polyplexes at a C/P ratio of 8:1, which was around 5-fold higher than CS polyplexes. The hIL-12 level for CS polyplexes was the lowest among all of the polyplexes at C/P ratios of 0.5:1 to 8:1. Interestingly, the level of IL-12 production increased with the HTCS polyplexes compared with PEI-based complexes at all C/P ratios in MCF-7 cells. The same elevation was observed in the HepG2 cell line at C/P ratios of 4:1 and 8:1. The level of hIL-12 production following the transfection of the cells with HTCS polyplexes at a C/P ratio of 8:1 was around 4.8- and 2.2-fold higher than with CS and PEI polyplexes, respectively.

Various strategies have been suggested to enhance the transfection efficiency of polycationic compounds, including the improvement of the buffering capacity [59,60], the modulation of charge density [61], and a reduction in toxicity [61]. The most probable reason for the high transfection efficiency of HTCS is the presence of positively charged groups, which could enhance its zeta potential and promote its condensation ability, resulting in the protection of plasmid DNA against degradation. The increase in cationic charge resulted in higher toxicity in both cell lines at all concentrations. However, lower concentrations can be used for polyplex formation in which the conjugate has no toxic effects. In other words, the concentrations used for polyplex formation are in the range of non-toxic concentrations. Overall, the improvement of transfection efficiency results in a balance between different factors, including condensation ability, particle size, and zeta potential, as well as toxicity and the induction of early escape from endosomes by the proton sponge effect.

## 4. Conclusions

Intratumoral injection of nucleic acid therapeutics, including plasmid-encoding cytokine genes, has been considered a promising strategy to localize the therapeutic effects while reducing systemic adverse reactions. Such therapeutic strategies need cytocompatible and efficient gene carriers enabling the delivery of oligonucleotides to the site of action. Hence, we developed a gene carrier based on *N*-[(2-hydroxy-3-trimethylammonium) propyl] chitosan salt (HTCS) for the delivery of a plasmid encoding the IL-12 gene. Our findings indicate that the quaternization of chitosan led to higher transfection efficiency of HTCS compared with unmodified chitosan and polyethyleneimine (PEI). This modification led to the formation of polyplexes with a desirable particle size of around 75 nm, which allows their injection via different routes of administration, including intravenous as well as intratumoral injections. The preparation of polyplexes based on a cytocompatible, water-soluble, and cost-effective material such as chitosan with a transfection efficiency higher than the gold standard of PEI may open up new horizons for the bench-to-bed translation of polymeric delivery systems. Further steps towards the clinical application of such materials include the evaluation of systemic and local toxic effects of the modified chitosan-based nanoparticles, as well the measurement of their gene transfection efficiency in animals. Considering the breakthrough discovery of mRNA as a novel therapeutic modality, this carrier system can also be tested for mRNA delivery.

## Figures and Tables

**Table 1:** Particle size and zeta potential of polyplexes formed with PEI (as a control), CS, and HTCS in HBG buffer at a C/P ratio of 8:1.

Polymer	Size by Intensity ± SD (nm)	Size by Volume ± SD (nm)	Size by Number ± SD (nm)	Zeta Potential ± SD (mV)
PEI	57.1 ± 0.6	68.8 ± 0.1	97.5 ± 0.4	+16.3 ± 1.6
CS	52.0 ± 0.2	77.8 ± 2.6	66.5 ± 0.5	+8.7 ± 0.6
HTCS	67.9 ± 0.2	89.45 ± 0.55	74.5 ± 0.7	+15.6 ± 1.1

## Data Availability

Not applicable.

## References

[1] Roma-Rodrigues C., Rivas-Garcia L., Baptista P.V., Fernandes A.R. (2020). Gene Therapy in Cancer Treatment: Why Go Nano?. Pharmaceutics.

[2] Kulkarni J.A., Witzigmann D., Thomson S.B., Chen S., Leavitt B.R., Cullis P.R., van der Meel R. (2021). The current landscape of nucleic acid therapeutics. Nat. Nanotechnol..

[3] Nguyen K.G., Vrabel M.R., Mantooth S.M., Hopkins J.J., Wagner E.S., Gabaldon T.A., Zaharoff D.A. (2020). Localized Interleukin-12 for Cancer Immunotherapy. Front. Immunol..

[4] Watkins S.K., Egilmez N.K., Suttles J., Stout R.D. (2007). IL-12 rapidly alters the functional profile of tumor-associated and tumor-infiltrating macrophages in vitro and in vivo. J. Immunol..

[5] Algazi A.P., Twitty C.G., Tsai K.K., Le M., Pierce R., Browning E., Hermiz R., Canton D.A., Bannavong D., Oglesby A. (2020). Phase II Trial of IL-12 Plasmid Transfection and PD-1 Blockade in Immunologically Quiescent Melanoma. Clin. Cancer Res..

[6] Halin C., Rondini S., Nilsson F., Berndt A., Kosmehl H., Zardi L., Neri D. (2002). Enhancement of the antitumor activity of interleukin-12 by targeted delivery to neovasculature. Nat. Biotechnol..

[7] Pishavar E., Oroojalian F., Ramezani M., Hashemi M. (2020). Cholesterol-conjugated PEGylated PAMAM as an efficient nanocarrier for plasmid encoding interleukin-12 immunogene delivery toward colon cancer cells. Biotechnol. Prog..

[8] Khalvati B., Sheikhsaran F., Sharifzadeh S., Kalantari T., Behbahani A.B., Jamshidzadeh A., Dehshahri A. (2017). Delivery of plasmid encoding interleukin-12 gene into hepatocytes by conjugated polyethylenimine-based nanoparticles. Artif. Cells Nanomed. Biotechnol..

[9] Hewitt S.L., Bailey D., Zielinski J., Apte A., Musenge F., Karp R., Burke S., Garcon F., Mishra A., Gurumurthy S. (2020). Intratumoral IL12 mRNA Therapy Promotes TH1 Transformation of the Tumor Microenvironment. Clin. Cancer Res..

[10] Sheikhsaran F., Sadeghpour H., Khalvati B., Entezar-Almahdi E., Dehshahri A. (2017). Tetraiodothyroacetic acid-conjugated polyethylenimine for integrin receptor mediated delivery of the plasmid encoding IL-12 gene. Coll. Surf. B Biointerfaces.

[11] Mohammadinejad R., Dehshahri A., Sagar Madamsetty V., Zahmatkeshan M., Tavakol S., Makvandi P., Khorsandi D., Pardakhty A., Ashrafizadeh M., Ghasemipour Afshar E. (2020). In vivo gene delivery mediated by non-viral vectors for cancer therapy. J. Control. Release.

[12] Ke L.J., Cai P.Q., Wu Y.L., Chen X.D. (2020). Polymeric Nonviral Gene Delivery Systems for Cancer Immunotherapy. Adv. Ther..

[13] Boussif O., Lezoualch F., Zanta M.A., Mergny M.D., Scherman D., Demeneix B., Behr J.P. (1995). A Versatile Vector for Gene and Oligonucleotide Transfer into Cells in Culture and in-Vivo-Polyethylenimine. Proc. Natl. Acad. Sci. USA.

[14] Dehshahri A., Oskuee R.K., Ramezani M. (2012). Plasmid DNA delivery into hepatocytes using a multifunctional nanocarrier based on sugar-conjugated polyethylenimine. Gene Ther. Mol. Biol..

[15] Santhakumaran L.M., Thomas T., Thomas T.J. (2004). Enhanced cellular uptake of a triplex-forming oligonucleotide by nanoparticle formation in the presence of polypropylenimine dendrimers. Nucleic Acids Res..

[16] Dehshahri A., Sadeghpour H., Oskuee R.K., Fadaei M., Sabahi Z., Alhashemi S.H., Mohazabieh E. (2014). Interleukin-12 plasmid DNA delivery using L-thyroxine-conjugated polyethylenimine nanocarriers. J. Nanopart. Res..

[17] Chuan D., Jin T., Fan R., Zhou L., Guo G. (2019). Chitosan for gene delivery: Methods for improvement and applications. Adv. Colloid Interface Sci..

[18] Madamsetty V.S., Tavakol S., Moghassemi S., Dadashzadeh A., Schneible J.D., Fatemi I., Shirvani A., Zarrabi A., Azedi F., Dehshahri A. (2021). Chitosan: A versatile bio-platform for breast cancer theranostics. J. Control. Release.

[19] Cao Y., Tan Y.F., Wong Y.S., Liew M.W.J., Venkatraman S. (2019). Recent Advances in Chitosan-Based Carriers for Gene Delivery. Mar. Drugs.

[20] Martins G.O., Petronio M.S., Lima A.M.F., Martinez A.M., Tiera V.A.D., Calmon M.D., Vilamaior P.S.L., Han S.W., Tiera M.J. (2019). Amphipathic chitosans improve the physicochemical properties of siRNA-chitosan nanoparticles at physiological conditions. Carbohydr. Polym..

[21] Strand S.P., Lelu S., Reitan N.K., de Lange Davies C., Artursson P., Varum K.M. (2010). Molecular design of chitosan gene delivery systems with an optimized balance between polyplex stability and polyplex unpacking. Biomaterials.

[22] Mazancova P., Nemethova V., Trelova D., Klescikova L., Lacik I., Razga F. (2018). Dissociation of chitosan/tripolyphosphate complexes into separate components upon pH elevation. Carbohydr. Polym..

[23] Balan V., Verestiuc L. (2014). Strategies to improve chitosan hemocompatibility: A review. Eur. Polym. J..

[24] Mansouri S., Lavigne P., Corsi K., Benderdour M., Beaumont E., Fernandes J.C. (2004). Chitosan-DNA nanoparticles as non-viral vectors in gene therapy: Strategies to improve transfection efficacy. Eur. J. Pharm. Biopharm..

[25] Lara-Velazquez M., Alkharboosh R., Norton E.S., Ramirez-Loera C., Freeman W.D., Guerrero-Cazares H., Forte A.J., Quinones-Hinojosa A., Sarabia-Estrada R. (2020). Chitosan-Based Non-viral Gene and Drug Delivery Systems for Brain Cancer. Front. Neurol..

[26] Park J.H., Saravanakumar G., Kim K., Kwon I.C. (2010). Targeted delivery of low molecular drugs using chitosan and its derivatives. Adv. Drug Deliv. Rev..

[27] Zubareva A., Shagdarova B., Varlamov V., Kashirina E., Svirshchevskaya E. (2017). Penetration and toxicity of chitosan and its derivatives. Eur. Polym. J..

[28] Jiang H.L., Xing L., Luo C.Q., Zhou T.J., Li H.S., Cho C.S. (2018). Chemical Modification of Chitosan as a Gene Transporter. Curr. Org. Chem..

[29] Xiao B., Wan Y., Wang X., Zha Q., Liu H., Qiu Z., Zhang S. (2012). Synthesis and characterization of N-(2-hydroxy)propyl-3-trimethyl ammonium chitosan chloride for potential application in gene delivery. Coll. Surf. B Biointerfaces.

[30] Chen K.Y., Zeng S.Y. (2018). Fabrication of Quaternized Chitosan Nanoparticles Using Tripolyphosphate/Genipin Dual Cross-Linkers as a Protein Delivery System. Polymers.

[31] Wang T.W., Xu Q., Wu Y., Zeng A.J., Li M.J., Gao H.X. (2010). Quaternized Chitosan (QCS) Nanoparticles as a Novel Delivery System for Ammonium Glycyrrhizinate. J. Nanosci. Nanotechnol..

[32] Heydari A., Dusicka E., Micusik M., Sedlak M., Lacik I. (2021). Unexpected counterion exchange influencing fundamental characteristics of quaternary ammonium chitosan salt. Polymer.

[33] Wang Y.Q., Wu J., Fan Q.Z., Zhou M., Yue Z.G., Ma G.H., Su Z.G. (2014). Novel vaccine delivery system induces robust humoral and cellular immune responses based on multiple mechanisms. Adv. Healthc. Mater..

[34] Wang Y.Q., Fan Q.Z., Liu Y., Yue H., Ma X.W., Wu J., Ma G.H., Su Z.G. (2016). Improving adjuvanticity of quaternized chitosan-based microgels for H5N1 split vaccine by tailoring the particle properties to achieve antigen dose sparing effect. Int. J. Pharm..

[35] Verheul R.J., Amidi M., van der Wal S., van Riet E., Jiskoot W., Hennink W.E. (2008). Synthesis, characterization and *in vitro* biological properties of O-methyl free N,N,N-trimethylated chitosan. Biomaterials.

[36] Peng Z.X., Wang L., Du L., Guo S.R., Wang X.Q., Tang T.T. (2010). Adjustment of the antibacterial activity and biocompatibility of hydroxypropyltrimethyl ammonium chloride chitosan by varying the degree of substitution of quaternary ammonium. Carbohydr. Polym..

[37] Dehshahri A., Sadeghpour H., Mohazzabieh E., Saatchi Avval S., Mohammadinejad R. (2020). Targeted double domain nanoplex based on galactosylated polyethylenimine enhanced the delivery of IL-12 plasmid. Biotechnol. Prog..

[38] Lee M., Nah J.-W., Kwon Y., Koh J.J., Ko K.S., Kim S.W. (2001). Water-soluble and low molecular weight chitosan-based plasmid DNA delivery. Pharm. Res..

[39] Dehshahri A., Alhashemi S.H., Jamshidzadeh A., Sabahi Z., Samani S.M., Sadeghpour H., Mohazabieh E., Fadaei M. (2013). Comparison of the effectiveness of polyethylenimine, polyamidoamine and chitosan in transferring plasmid encoding interleukin-12 gene into hepatocytes. Macromol. Res..

[40] Hallaj-Nezhadi S., Valizadeh H., Dastmalchi S., Baradaran B., Jalali M.B., Dobakhti F., Lotfipour F. (2011). Preparation of chitosan-plasmid DNA nanoparticles encoding interleukin-12 and their expression in CT-26 colon carcinoma cells. J. Pharm. Pharm. Sci..

[41] Lapitsky Y., Zahir T., Shoichet M.S. (2008). Modular biodegradable biomaterials from surfactant and polyelectrolyte mixtures. Biomacromolecules.

[42] Richard I., Thibault M., De Crescenzo G., Buschmann M.D., Lavertu M. (2013). Ionization behavior of chitosan and chitosan-DNA polyplexes indicate that chitosan has a similar capability to induce a proton-sponge effect as PEI. Biomacromolecules.

[43] Domard A. (1987). pH and c.d. measurements on a fully deacetylated chitosan: Application to Cu(II)-polymer interactions. Int. J. Biol. Macromol..

[44] Vermeulen L.M.P., De Smedt S.C., Remaut K., Braeckmans K. (2018). The proton sponge hypothesis: Fable or fact?. Eur. J. Pharm. Biopharm..

[45] Wang C., Huang X., Sun L., Li Q., Li Z., Yong H., Che D., Yan C., Geng S., Wang W. (2022). Cyclic poly(β-amino ester)s with enhanced gene transfection activity synthesized through intra-molecular cyclization. Chem. Commun..

[46] Ma P.L., Lavertu M., Winnik F.M., Buschmann M.D. (2017). Stability and binding affinity of DNA/chitosan complexes by polyanion competition. Carbohydr. Polym..

[47] Schaffer D.V., Fidelman N.A., Dan N., Lauffenburger D.A. (2000). Vector unpacking as a potential barrier for receptor-mediated polyplex gene delivery. Biotechnol. Bioeng..

[48] Nimesh S., Aggarwal A., Kumar P., Singh Y., Gupta K.C., Chandra R. (2007). Influence of acyl chain length on transfection mediated by acylated PEI nanoparticles. Int. J. Pharm..

[49] Oskuee R.K., Dehshahri A., Shier W.T., Ramezani M. (2009). Alkylcarboxylate grafting to polyethylenimine: A simple approach to producing a DNA nanocarrier with low toxicity. J. Gene Med..

[50] Ogris M., Steinlein P., Kursa M., Mechtler K., Kircheis R., Wagner E. (1998). The size of DNA/transferrin-PEI complexes is an important factor for gene expression in cultured cells. Gene Ther..

[51] Xiang S., Tong H., Shi Q., Fernandes J.C., Jin T., Dai K., Zhang X. (2012). Uptake mechanisms of non-viral gene delivery. J. Control. Release.

[52] Chen J., Wang K., Wu J., Tian H., Chen X. (2019). Polycations for Gene Delivery: Dilemmas and Solutions. Bioconjug. Chem..

[53] Gan Q., Wang T., Cochrane C., McCarron P. (2005). Modulation of surface charge, particle size and morphological properties of chitosan-TPP nanoparticles intended for gene delivery. Coll. Surf. B Biointerfaces.

[54] Martin V., Ribeiro I.A.C., Alves M.M., Goncalves L., Almeida A.J., Grenho L., Fernandes M.H., Santos C.F., Gomes P.S., Bettencourt A.F. (2019). Understanding intracellular trafficking and anti-inflammatory effects of minocycline chitosan-nanoparticles in human gingival fibroblasts for periodontal disease treatment. Int. J. Pharm..

[55] Thanh V.M., Nguyen T.H., Tran T.V., Ngoc U.P., Ho M.N., Nguyen T.T., Chau Y.N.T., Le V.T., Tran N.Q., Nguyen C.K. (2018). Low systemic toxicity nanocarriers fabricated from heparin-mPEG and PAMAM dendrimers for controlled drug release. Mater. Sci. Eng. C Mater. Biol. Appl..

[56] Moghimi S.M., Symonds P., Murray J.C., Hunter A.C., Debska G., Szewczyk A. (2005). A two-stage poly(ethylenimine)-mediated cytotoxicity: Implications for gene transfer/therapy. Mol. Ther..

[57] Jain K., Kesharwani P., Gupta U., Jain N.K. (2010). Dendrimer toxicity: Let’s meet the challenge. Int. J. Pharm..

[58] Monnery B.D., Wright M., Cavill R., Hoogenboom R., Shaunak S., Steinke J.H.G., Thanou M. (2017). Cytotoxicity of polycations: Relationship of molecular weight and the hydrolytic theory of the mechanism of toxicity. Int. J. Pharm..

[59] Liu M.C., Hu Y.L., Feng Y. (2020). Evaluation of low molecular weight polyethylenimine-introduced chitosan for gene delivery to mesenchymal stem cells. Mater. Express.

[60] Jiang H.L., Kim Y.K., Arote R., Nah J.W., Cho M.H., Choi Y.J., Akaike T., Cho C.S. (2007). Chitosan-graft-polyethylenimine as a gene carrier. J. Control. Release.

[61] Li Z.T., Guo J., Zhang J.S., Zhao Y.P., Lv L., Ding C., Zhang X.Z. (2010). Chitosan-graft-polyethylenimine with improved properties as a potential gene vector. Carbohydr. Polym..

